# White matter neural substrates in alcohol dependence with genetic risk and their role in pathological reward process

**DOI:** 10.1038/s41598-025-18003-z

**Published:** 2025-09-26

**Authors:** Fei Wu, Guowei Wu, Ping Dong, Jiahui Deng, Xuejiao Gao, Peng Li, Junliang Yuan, Hongqiang Sun

**Affiliations:** 1 NHC Key Laboratory of Mental Health (Peking University), Peking University Sixth Hospital, Peking University Institute of Mental Health, National Clinical Research Center for Mental Disorders (Peking University Sixth Hospital), Peking University, Beijing, 100191 China; 2 CAS Key Laboratory of Behavioral Science, Institute of Psychology, Chinese Academy of Sciences, Beijing, China; 3 Department of Neurology, NHC Key Laboratory of Mental Health (Peking University), Peking University Sixth Hospital, Peking University Institute of Mental Health, National Clinical Research Center for Mental Disorders (Peking University Sixth Hospital), Peking University, Beijing, 100191 China

**Keywords:** Alcohol dependence, Reward system, Genetic risk, White matter microstructure, Topological organization, Psychology, Medical research

## Abstract

Studies have revealed significant evidence of white matter (WM) microstructural and network connectome abnormalities in alcohol use disorder (AUD). However, the neuroimaging characteristics of alcohol dependence (AD) patients with a family history of AUD and the role of these changes in pathological craving remain unclear. Investigating the heritability of AUD is crucial for identifying genetic predispositions and informing targeted prevention strategies. The study recruited 51 patients for AD, 21 patients with a family history positive (FHP), 30 patients with a family history negative (FHN), and 25 healthy controls (HC). We compared fractional anisotropy (FA) and mean diffusivity (MD) of striatal circuits and topological properties of the reward system between the three groups. Then, covariates of alcohol use characteristics (duration and severity of AD) were controlled between FHP and FHN. We found abnormal topological properties of hippocampus in AD with FHP compared to HC. After controlling for covariates, there were still disruptions of topology organization in FHP compared to FHN, such as lower nodal betweenness, nodal degree and higher shortest path of right hippocampus. The nodal topological properties of right hippocampus were significantly correlated with self-reported craving in AD. Our findings provide robust evidence for WM neural abnormalities in AD with high genetic risk. We also found the disrupted topological properties of the right hippocampus associated with craving level.

**Supplementary Information:**

The online version contains supplementary material available at 10.1038/s41598-025-18003-z.

## Introduction

Alcohol consumption has been widely prevalent in the world^1,2^. Its abuse can become pathological, leading to the development of alcohol use disorder (AUD) and even more severe stages such as alcohol dependence (AD)^3^. Many disease and mortality outcomes are impacted causally by alcohol consumption, such as cancers, cardiovascular diseases, neuropsychiatric disorders and accidents, and most often in an accelerated dose–response manner^4–6^. Relapse and a poor prognosis remain the primary challenges for the prevention and treatment of AD^7^. Several variables, such as the presence of high genetic risk (family history positive for AUD), which may influence intervention effectiveness, have not always been considered^8^. Therefore, it is necessary to investigate biomarkers or treatment targets of high genetic risk subtype of AD.

AUD is to a high degree heritable, with a heritability of approximately 50%^9^. It is standard procedure to examine family history (FH) while evaluating AUD patients in clinical settings. Such details may be crucial for a diagnosis and treatment strategy. Based on the findings from whole genome-transcriptomic analysis, the identification of heritable gene networks enables targeted interventions for AD by informing personalized therapeutic strategies tailored to individual genetic vulnerabilities^10^. A positive family history (FHP) is a vulnerability factor that may increase the risk of developing AUD and augments the severity and pathological features of alcohol use- related disease in offspring^11,12^. A positive family history (FHP) is a vulnerability factor that may increase the risk^13–15^. A multisite study found that FHP of AUD was associated with self-rated impulsivity as assessed by the Barratt Impulsiveness Scale (BIS) in a clinical sample of AUD patients^14^. The result suggested that FHP within AUD patients could be a marker of higher impulsivity. A previous study investigated potential cognitive endophenotypes in AD and discovered AD patients with FHP had significantly lower intelligence quotient scores than family history negative (FHN)^15^. Studies have also shown that disturbed sleep is a risk factor for developing AD, and that FHP is a unique risk factor for sleep complaints among individuals with AD^16^. FHP may indeed be associated with different clinical characteristics, and could be a marker of a clinically distinct subgroup of AD patients in need of specific clinical care^8,14^.

Brain white matter (WM) structure injuries are characteristic impairment of AD. WM structures efficiently propagate neural signals between spatially distinct cortical regions, thereby improving brain functional connectivity^17,18^. Several processes might account for neurodegeneration in AD, such as the toxic effects of alcohol and its metabolites itself as well as the frequent co-existing nutritional or vitamin deficiencies. Thus, chronic alcohol use causes extensive disruption to WM microstructure, primarily resulting from demyelination, microtubule disruption and axonal loss^19,20^. Disruption of WM may degrade neural signal transmission and the capacity for certain cognitive functions, resulting in enhanced impulsivity, poor inhibitory control, working memory performance and reduction in cognitive flexibility^21,22^. These cognitive deficits reflect a possible breakdown of WM structures connecting regions in charge of executive processing. Damage or demyelination along neuronal axons usually manifests in lower fractional anisotropy (FA) and/or higher mean diffusiv Based on the findings ity (MD) values^23,24^. Given that WM structure and connectivity coexist, the brain network as a whole is also critical for understanding AD susceptible neurobiology^25,26^. Applying graph theory to WM structure network suggested that WM network was organized to allow optimized efficiency, such as that observed in small-world and global efficiency^27^. Cortico-limbic-striatal brain networks are responsible for cognitive tasks related to reward processing, inhibition control, and monitoring in AD^28^. As the memory center, hippocampus promotes pathological memories associated with alcohol and mediates cue-induced reinstatement of alcohol-seeking behavior^29,30^. Topological structure alterations in nodes of the reward system, particularly striatum and hippocampus might provide insight into endophenotypes of AD^31^.

Several studies have explored structural and functional brain network vulnerability in substance-naive immediate relatives of AUD^32–34^. A brain graph study comparing resting brain functional connectivity in substance-naive high-risk (HR) male offspring and matched healthy low-risk (LR) males. They found reduced clustering, small-worldness and local network efficiency in HR with FHP of AUD compared to controls^35^. Another structural network study examined the whole-brain WM local topology of reward system in patients with AUD and unaffected siblings relative to healthy controls. They found both nodal clustering coefficient and nodal local efficiency rank ordered (Control > Sibling > AUD) in reward system nodes^31^. WM connectome was used in a recent study to compare adult FHP and FHN groups, FHP group showed decreased major tract connectivity in cortico-striatal pathway, left cortico-thalamic pathway etc^36^. The aforementioned studies suggest suggested that there are premorbid differences in brain structure and function in FHP individuals without substance abuse relative to their FHN peers. WM impairments are exacerbated by chronic alcohol exposure and may be mediated through heritable traits^37^. However, whether AD patients with genetic risk specifically alters the neural substrates of WM microstructure and topological networks remains unclear.

The aim of the current study was to investigate potential neuro-substrates in AD with high genetic risk and to provide a theoretical basis for targeted intervention. First, WM integrity of striatal circuits and the topological structure of reward systems (caudate, putamen and hippocampus) were compared in AD with FHP, FHN and HC. Then white matter structure connection and topology with FHP and FHN were compared by controlling alcohol use related confounding factors. Finally, the correlations were explored between the abnormal white matter network indexes and self-reported craving.

## Materials and methods

### Subjects and procedure

A total of 51 male patients meeting DSM-IV criteria of AD from the psychiatric ward of Peking university sixth hospital were enrolled in the study, consisting of 21 with FHP and 30 with FHN. These hospitalized AD patients had all completed acute withdrawal and maintained 2–4 weeks of supervised abstinence prior to study participation. For comparison, we recruited 25 healthy controls (HC) through community advertisements in Beijing, who underwent rigorous screening to confirm the absence of personal or family history of substance use disorders. The HC group was matched to the AD groups for age, education level, and gender distribution to ensure appropriate comparability. The definition of FHP in our study is individuals who had at least one biological parent or two or more second-degree relatives diagnosed with AUD or problem drinking, while FHN individuals had an absence of familial substance use disorder in first or first and second-degree relatives^38^. All 51 AD patients met the DSM-Ⅳ criteria of AD and had finished acute withdrawal with abstinence for at least two weeks. All patients exhibited a Clinical Institute Withdrawal Assessment for Alcohol-Revised (CIWA-Ar) score < 7. Meanwhile, a total number of 25 age-, education- and gender-matched participants without FHP were recruited as HC. All subjects were male and right-handed.

Clinical interviews were conducted on AD by an expert clinician about their drinking history using self-made questionnaires and the Michigan Alcoholism Screening Test (MAST). Mental states and impulsive traits were also assessed using instruments such as the Self-Rating Anxiety Scale (SAS), Self-Rating Depression Scale (SDS), and the 11th version of the Barratt Impulsiveness Scale (BIS-11). AD participants were requested to evaluate the intensity of their subjective craving for alcohol consumption on a 100-point Visual Analogue Scale (VAS) ranging from 0 (not at all) to 100 (extremely high). All HC underwent identical clinical assessments as AD participants, with the exception of CIWA-Ar and craving scales. The study protocol was approved by the Ethics Committee of Peking University Sixth Hospital, and all participants completed informed consent.

The inclusion criteria of AD in our study were: (1) Male, aged 18–60, right-handed. (2) Meet the DSM-IV diagnostic criteria for alcohol dependence. (3) The score of the CIWA-Ar was less than 7. Exclusion criteria: (1) The presence of a relevant axis I disorder such as schizophrenia, bipolar disorder, depression, generalized anxiety disorder, and other DSM-IV Axis I diagnosed psychiatric disorders (except nicotine dependence). (2) Clinically significant comorbidities involving major organ systems (e.g., cardiac, hepatic, renal) or progressive neurological disorders (e.g., cerebrovascular disease, neurodegenerative conditions, moderate-severe traumatic brain injury) were exclusion criteria. (3) There are contraindications to magnetic resonance examination: metallic implants (e.g., pacemakers, aneurysm clips); suspected ferromagnetic foreign bodies; and claustrophobia requiring sedation. The demographic characteristics of participants are shown in (Table 1). The HC participants were recruited from the community and had never met DSM-IV criteria for AD or any other DSM-IV Axis I disorder.

**Table 1:** Baseline demographics and the clinical characteristics.

Variables	HC (*N* = 25)	FHN (*N* = 30)	FHP (*N* = 21)	F/Z/T	*p* value
Age (y)	41.44 ± 9.94	43.13 ± 8.03	41.43 ± 7.26	0.361	0.698
Education (y)	13.00 (9.50,16.00)	12.00 (8.75,15.00)	12.00 (9.50,15.00)	0.740	0.697
Duration of ethanol intake (y)	NA	24.83 ± 7.55	23.05 ± 6.97	0.857	0.395
Duration of dependence (y)	NA	5.50 (3.00,10.00)	5.00 (3.75,8.00)	-0.164	0.870
Abstinence (day)	NA	21.00 (14.00,28.00)	21.00 (14.00,29.00)	-0.464	0.642
CIWA-Ar	NA	1.00 (0.00,3.00)	2.00 (1.00,3.00)	-1.050	0.294
MAST	0.32 ± 1.22	12.43 ± 3.79 ^a^	15.05 ± 4.24 ^b, c^	135.028	< 0.001
C-VAS	NA	0.00	0.00	-0.380	0.704
		(0.00,0.00)	(0.00,2.00)		
SDS	32.36 ± 8.563	37.07 ± 12.275	37.62 ± 11.215	1.738	0.183
SAS	30.00 (26.00,35.00)	32.00 (27.00,41.00)	36.00 (28.75,44.50)	5.924	0.052
BIS-11	49.56 ± 10.716	52.20 ± 12.57	55.57 ± 13.97	1.340	0.268

Abbreviations: CIWA-Ar, Alcohol Withdrawal Syndrome Scale; MAST, Michigan alcoholism screening test; C-VAS, Craving-Visual Analog Scale; SAS, Self-rating anxiety scale; SDS, Self-rating depression scale; BIS-11, Barratt impulsiveness scale, 11th version.

^a^ Significant post hoc differences between the HC and FHN with *p* < 0.05.

^b^ Significant post hoc differences between the HC and FHP with *p* < 0.05.

^c^ Significant post hoc differences between the FHN and FHP with *p* < 0.05.

### MRI data acquisition

The study was conducted in Peking University Sixth Hospital using the GE Discovery MR750 3.0T. Three-dimensional (3D) T1-weighted images were obtained using an MPRAGE pulse sequence, with a voxel size of 1 × 1 × 1 mm^3^ (repetition time [TR] = 6.7 ms; echo time [TE] = 2.9 ms; data matrix = 240 × 240mm^2^; slices = 170; field of view [FOV] = 240 × 240 mm^2^. Diffusion tensor imaging (DTI) data were collected using a single-shot echo planar imaging (EPI) sequence, with a voxel size of 2 × 2 × 2mm^3^ (TR = 8900ms, TE = 92 ms, data matrix = 120 × 120, FOV = 240 × 240 mm^2^slice = 72). To enhance the signal-to-noise ratio, 64 repetitions of the 32 non-collinear directions (b = 1000 s/mm^2^) were applied, along with 8 acquisitions without diffusion weighting (b = 0 s/mm^2^).

### Striatum structural connectivity analysis based on probabilistic tractography

**Fig. 1:**
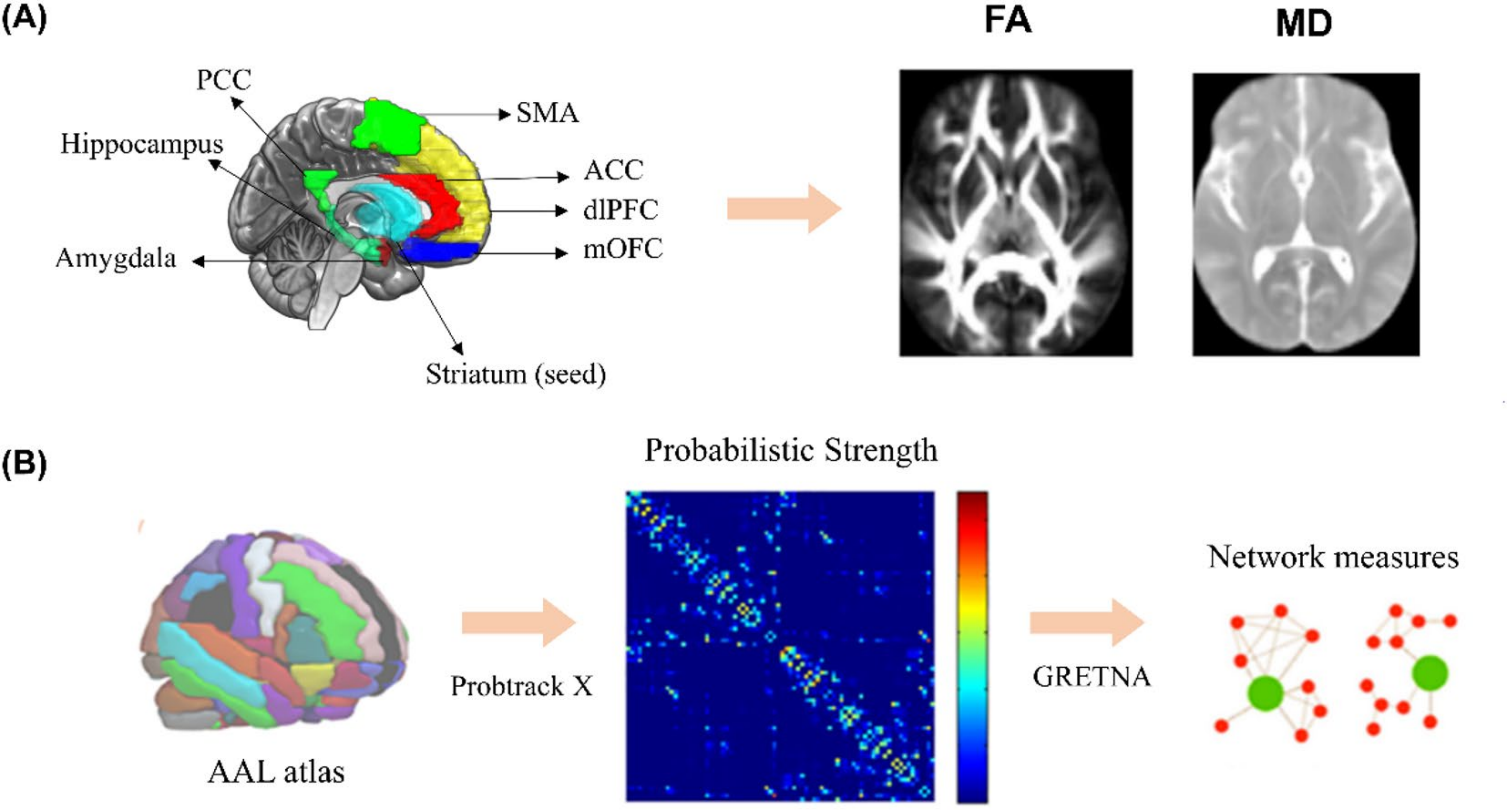
Target regions for seed-based tractography and constructions of brain network. (**A**) The seven target brain masks (five cortical cortex and two subcortical regions) for striatum tractography (**B**) Brain networks were constructed with the cortical and subcortical seeds representing its nodes.

Regions of interest (ROIs) were selected based on activation during cue reactivity tasks in previous fMRI studies related to reward network (Fig. 1A)^39–42^. The striatum seed was derived from a combination of regions in the standard Anatomical Automatic Labeling (AAL) template, which was transformed into individual subject diffusion space using a two-step normalization approach with T1 and FA images. To assess striatal connectivity with other regions, a set of masks consisting of five cortical regions and two subcortical regions was used. These masks were defined by previous study^43^and calculated separately for left and right using the standard AAL template, including the dorsolateral prefrontal cortex (dlPFC), medial orbitofrontal cortex (mOFC), supplementary motor area (SMA), anterior cingulate cortex (ACC), posterior cingulate cortex (PCC), hippocampus, and amygdala.

Probabilistic tractography by using the seed-based method was employed in this study to map specific circuits related to the striatum and target brain regions. FMRIB’s Diffusion Toolbox (FDT) was used to preprocess the diffusion image, including visual image quality checking, averaging of the 8 b0 images to calculate the mean b0 image, and eddy current correction to reduce distortions from EPI artefacts and motion correction. The FA and MD maps were then obtained through FDT. BEDPOSTX GPU version was used to calculate the distribution of fibre orientations at each brain voxel, and probtrackx GPU was used to initiate probabilistic tractography from each voxel within the seed ROI with specific parameters. The resulting tracts were binarized and masked with diffusion maps to compute the mean value of FA and MD within the final tract mask in diffusion space for each subject. Specifically, the maximum connectivity value was obtained with fslstats, and voxels with values of more than 5% of the maximum connectivity value were kept. To ensure that only white matter fibers were tracked, tractography for each hemisphere was completed separately, with the left (right) striatum as a seed mask and each target left (right) ROI as a termination mask. Additionally, the side hemisphere mask was used as an exclusion mask.

### Structure connectome creation

Interregional white matter connectivity was assessed using probabilistic tractography through FSL’s probtrackx2, which estimates the orientation of a tract by sampling from the principal diffusion direction calculated in bedpostx and generates a distribution of the tract’s path from each voxel. Multiple tracts are sampled from each voxel, and each propagation step is based on a randomly chosen orientation from the probability map. The probability of a tract starting at the seed region and passing through the target region is used to estimate connectivity between two regions.

To estimate connectivity between every ROI in the atlas, tractography was performed using the GPU version of probtrackx2. Parameters were set based on Kuhn^44^these parameters ensured that 5,000 sample tracts were generated from the center of each voxel of each ROI and only tracts that reached a target ROI and passed through white matter were retained.

90 nodes AAL templates used in this study, which resulted in a 90 × 90 connectivity matrix for each hemisphere, where each entry represented the streamline count between each pair of nodes (Fig. 1B). Streamline count covaries with both the number of axons connecting two regions and the microstructural integrity of those axons. The diagonal elements of the matrices represent self-connections and were excluded from analyses. To account for variability related to differences in ROI size within and across individuals and differences in the ability of tractography to reconstruct different white matter pathways, the retained streamline counts originating from each seed ROI were normalized by the total number of tracts that were retained for that ROI. This resulted in values reflecting the proportion of streamlines originating from the seed ROI that connect to each of the other ROIs.

Due to the probabilistic nature of the tracking algorithm and potential non-reciprocal connections between regions, the number of streamlines originating from one region and terminating in another is not equivalent to the number originating from the latter and terminating in the former. This caused the upper and lower diagonals of the initial connectivity matrix to be non-symmetric. However, because diffusion MRI cannot detect directionality, the number of tracts from one seed region to another target region should be equivalent to that from the latter to the former. Thus, the matrices were symmetrized by averaging the number of tracts of the two matrix elements representing the same connection.

### Topological properties based on graph theory

In order to characterize the topology of the structural network on a global scale, various network properties were computed. These included the clustering coefficient, shortest path length, global efficiency, and local efficiency as described by Newman^45^. To assess the presence of small-world properties, normalized global measures of the shortest path length (λ) and clustering coefficient (γ) were computed, as well as the small-worldness parameter (σ), which is defined as σ = γ/λ. Global L rand p and Global C rand p were calculated as the mean values of the global clustering coefficient and global shortest path length, respectively, across 1000 random networks. Nodal-wise properties including nodal clustering coefficient (CC-nodal), nodal efficiency (E-nodal), nodal local efficiency (LE-nodal) and nodal shortest path (Sp-nodal).

All network properties were computed using the GRETNA toolbox, which is a graph theoretical network analysis tool designed specifically for imaging connectomes. To ensure that only reliable connections were included in the analysis, any connections that were present in less than 20% of the group subjects were excluded from the connectivity matrix prior to the calculation of the global network properties. For each subject, probabilistic tractography yielded a 90 × 90 streamline matrix. To equalise graph density across participants, we applied proportional thresholds from 0.05 to 0.15 in 0.025 increments, retaining the strongest 5–15% of edges^46^. Any connections with a weight below the chosen threshold were considered spurious and removed. To summarize the topological properties of the network, we calculated the area under the curve (AUC) for both global and local metrics. This allowed us to obtain a scalar value that was independent of a single threshold selection^47^. For node-wise metrics, we only choose left and right caudate, left and right putamen, left and right hippocampus as the interest nodes included in reward system. Benjamini and Hochberg false discovery rate (FDR-BH) correction was used in multiple comparisons.

### Statistical analysis

One-way analysis of variance (ANOVA) or Kruskal-Wallis Test was used to compare age, years of education and clinical variables among FHP, FHN and HC groups. For the DTI results, ANOVA was used to preliminarily compare the FA and MD of systematic striatal circuits and the topological organization of reward system (caudate, putamen and hippocampus) among the three groups. The FDR-BH method was used to correct the multiple comparisons. We also separately tested for ordered differences between the three groups by using the Jonckheere-Terpstra Test.

Considering age and alcohol clinical features are potential nuisances to diffusion parameters and topological organizations, the age, duration and severity (MAST) of AD were controlled as covariate variables in analysis of covariance between the FHP and FHN groups. To further examine the associations between the altered nodal metrics and subjective craving values. The altered nodal metrics were extracted, and then Spearman rank correlation analysis was used to evaluate the correlation between the nodal metrics and subjective craving (craving-VAS) in the AD group.

## Results

### Demographics and clinical characteristics of FHP, FHN and HC

The sample included a total of 21 FHP AD patients (mean age 41.43 ± 7.26 years), 30 FHN AD patients (mean age 43.13 ± 8.03 years) and 25 HC (mean age 41.44 ± 9.94 years). Age, sex and education were matched among the three groups (Table 1). FHP patients displayed significantly higher MAST scores compared to FHN and controls. (Table 1).

### Differences in structural connections of striatal tracts among FHP, FHN and HC

**Fig. 2:**
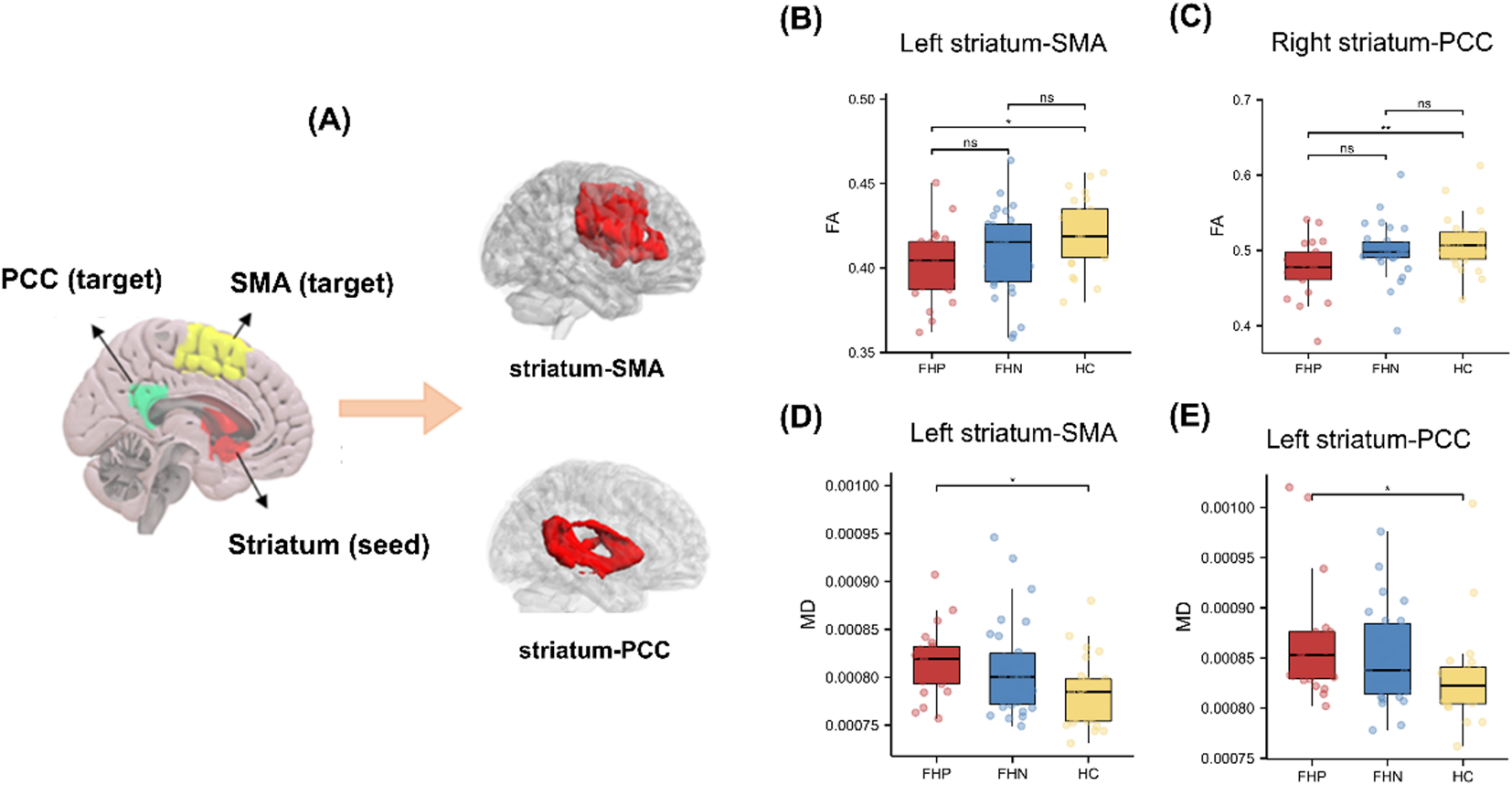
Decreased WM microstructure structural connectivity in AD patients with FHP compared with controls in striatal circuits. (**A**) WM structure connection of striatum to supplementary motor area (SMA) and posterior cingulate cortex (PCC). (**B**) The comparisons of FA values of left striatum-SMA among three groups (**C**) The comparisons of FA values of right striatum-PCC among three groups (**D**) The comparisons of MD values of left striatum-SMA among three groups (**E**) The comparisons of MD values of left striatum-PCC among three groups. *, ** indicated group differences at the *p* < 0.05, 0.01values, respectively. However, these effects did not survive correction for multiple comparisons.

One-way ANOVA revealed nominal significance for group differences in striatal microstructure, though some of these did not survive FDR-BH correction. These striatal circuits include left striatum-SMA (FA; MD) and right striatum-SMA (FA; MD), right striatum-PCC (FA) and left striatum-PCC (MD), left striatum-amygdala (FA), left striatum-ACC (MD) (Supplementary Tables 1 and 2). The post-hoc analysis demonstrated that the FA of right striatum-SMA and left striatum-amygdala, MD of right striatum-SMA and left striatum-ACC are disrupted in FHP and FHN compared with HC. The FA of left striatum-SMA and right striatum-PCC are lower, and the MD of left striatum-SMA and left striatum-PCC are higher in FHP compared with HC (Fig. 2). These effects did not survive correction for multiple comparisons.

### Differences in topological properties among FHP, FHN and HC

Between-group comparisons on global topological properties of WM structure brain networks showed there is no difference between the three groups. Specifically, no significant differences were observed in clustering coefficient, shortest path length, local efficiency, global efficiency and small-worldness (Table 2).

**Table 2:** Global metrics of brain WM structural networks of FHP, FHN and HC groups.

Topology properties	FHP (*N* = 21)	FHN (*N* = 30)	HC (*N* = 25)	F	*P*
Clustering Coefficient	0.140 ± 0.0008	0.140 ± 0.0008	0.139 ± 0.0027	1.031	0.362
Shortest Path Length	0.420 ± 0.001	0.430 ± 0.001	0.430 ± 0.010	1.136	0.327
Local Efficiency	0.189 ± 0.0004	0.189 ± 0.0004	0.189 ± 0.0015	1.024	0.364
Global Efficiency	0.137 ± 0.0003	0.137 ± 0.0002	0.490 ± 0.0032	1.047	0.356
Small-Worldness	0.461 ± 0.007	0.462 ± 0.009	0.469 ± 0.033	1.192	0.309

Abbreviations: FHP, family history positive; FHN, family history negative; HC, healthy controls.

**Fig. 3:**
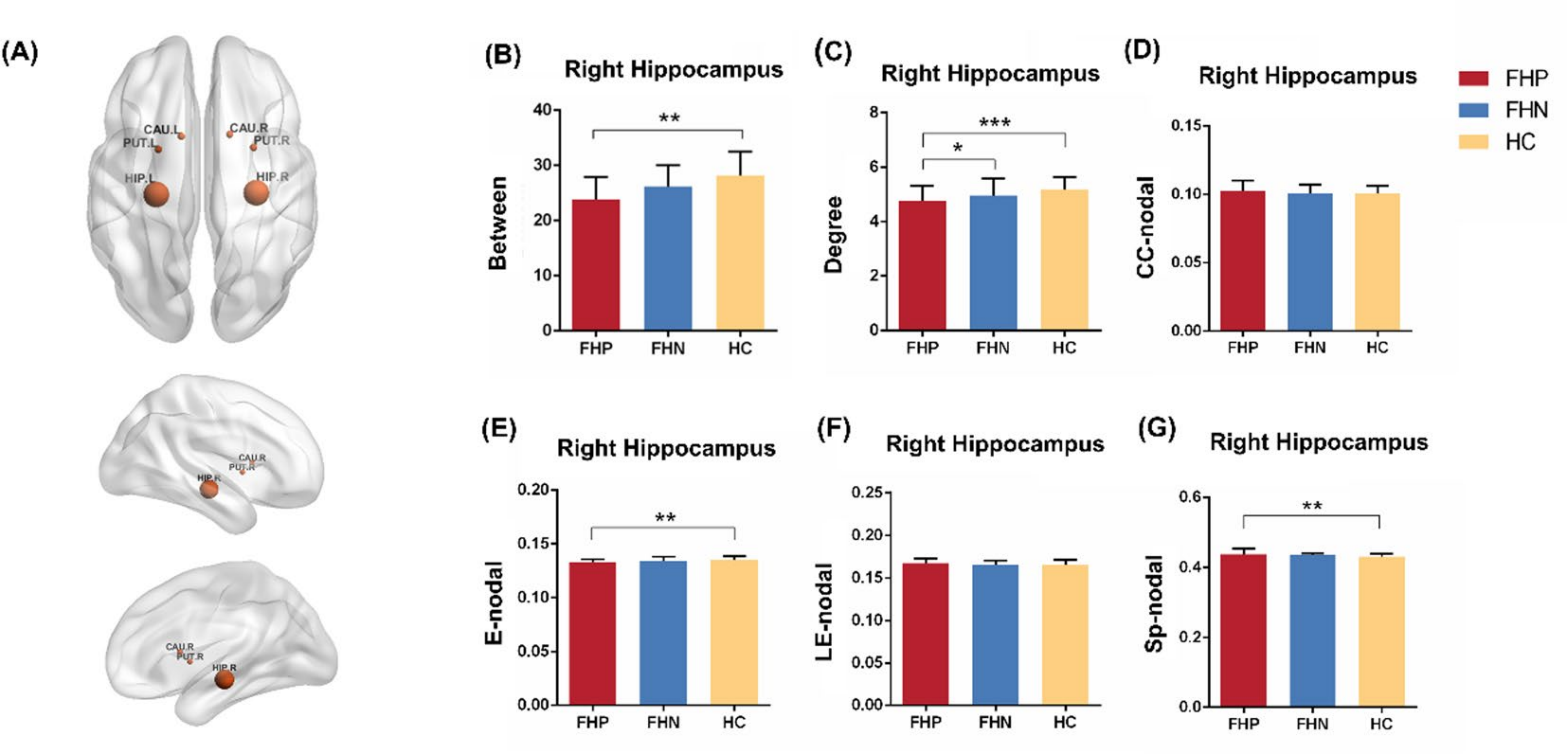
Comparisons of reward system nodal topological organization among FHP, FHN and HC. (**A**) Reward system specific node. (**B**) The comparisons of nodal betweenness of right hippocampus among three groups (**C**) The comparisons of nodal degree of right hippocampus among three groups (**D**) The comparisons of CC-nodal of right hippocampus among three groups (**E**) The comparisons of E-nodal of right hippocampus among three groups (**F**) The comparisons of LE-nodal of right hippocampus among three groups (**G**) The comparisons of Sp-nodal of right hippocampus among three groups. *, **, and *** indicated within group differences at the *p* < 0.05, 0.01, and 0.001 values, respectively.

Then, between-group comparisons were made on nodal topology of reward system, including the following brain regions: left and right caudate, left and right putamen, left and right hippocampus. FHP patients displayed significantly lower right hippocampus nodal betweenness, lower right hippocampus nodal degree, lower right hippocampus E nodal and higher right hippocampus Sp-nodal compared to controls, but not found in FHN (Fig. 3; Table 3). No significant differences between groups were found in nodal topology of left and right caudate, left and right putamen and left hippocampus (Supplementary Table 3), Jonckheere-Terpstra Testing also did not show significant ordered differences (Control > FHN > FHP or Control < FHN < FHP) in the three groups.

**Table 3:** Right hippocampus nodal topological organizations among FHP, FHN and HC groups.

Properties	FHP (*N* = 21)	FHN (*N* = 30)	HC (*N* = 25)	F	*P*	FDR-BH *p*
Betweenness	23.837 ± 4.051	26.165 ± 3.891	28.184 ± 4.301	6.490	0.003	**0.006**
	*P* = 0.150					
		*P* = 0.221				
	*P* = 0.002					
Degree	4.775 ± 0.320	4.974 ± 0.259	5.142 ± 0.270	9.810	< 0.001	**0.001**
	*P* = 0.046					
		*P* = 0.094				
	*P* < 0.001					
CC-nodal	0.102 ± 0.004	0.101 ± 0.003	0.101 ± 0.002	2.207	0.117	0.136
E-nodal	0.133 ± 0.002	0.134 ± 0.002	0.135 ± 0.002	5.956	0.004	**0.006**
	*P* = 0.174					
		*P* = 0.268				
	*P* = 0.003					
LE-nodal	0.167 ± 0.003	0.166 ± 0.002	0.166 ± 0.002	2.054	0.136	0.136
Sp-nodal	0.437 ± 0.006	0.434 ± 0.005	0.431 ± 0.008	6.044	0.004	**0.006**
	*P* = 0.150					
		*P* = 0.293				
	*P* = 0.003					

Abbreviations: FHP, family history positive; FHN, family history negative; HC, healthy controls; CC-nodal, Nodal clustering coefficient; E-nodal, Nodal efficiency; LE-nodal, Nodal local efficiency; Sp-nodal, Nodal Shortest path.

### Differences in WM tracts integrity and topological properties between FHP, FHN by controlling age and alcohol use clinical characters

**Fig. 4:**
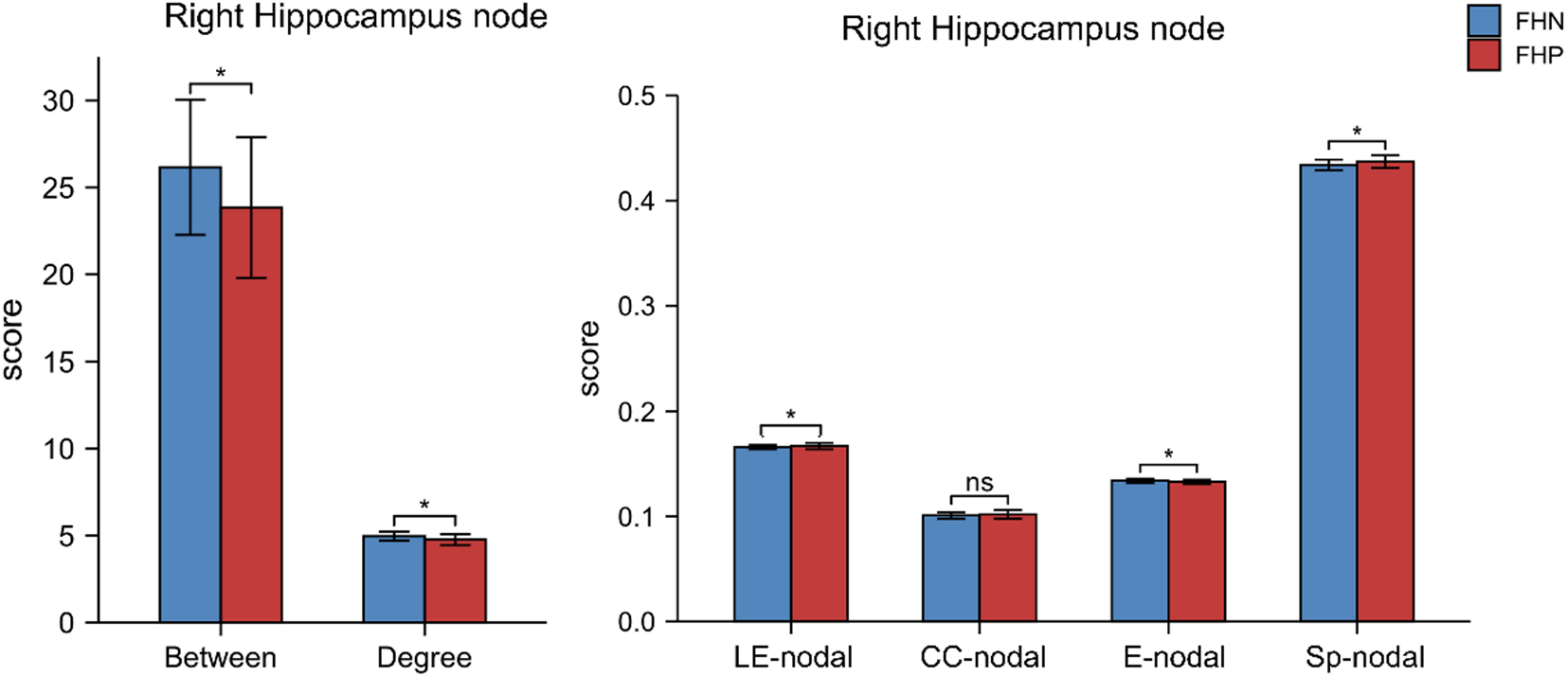
Altered nodal topological organization of right hippocampus in FHP compared with FHN (alcohol use clinical characters controlled as covariate variables). Abbreviations: FHP, family history positive; FHN, family history negative; CC-nodal, Nodal clustering coefficient; E-nodal, Nodal efficiency; LE-nodal, Nodal local efficiency; Sp-nodal, Nodal shortest path.

Considering the age and alcohol clinical characters may affect the WM microstructures and topological organizations, we controlled age and severity of AD and dependence courses as covariate variables in the analysis of covariance to compare the difference between FHP and FHN. No significant WM microstructure (FA and MD) differences between FHP and FHN groups were found in the striatal circuits after controlling the confounding factors (Supplementary Table 4). FHP patients displayed significantly lower right hippocampus nodal betweenness, lower right hippocampus nodal degree, lower right hippocampus E-nodal and higher right hippocampus LE-nodal, and higher right hippocampus Sp-nodal compared to controls (Fig. 4, Supplementary Table 5). No significant differences between FHP and FHN groups were found in nodal topology of left and right caudate, left and right putamen and left hippocampus (Supplementary Table 5).

### Relationships between right hippocampus nodal topological properties and clinical variables

**Fig. 5:**
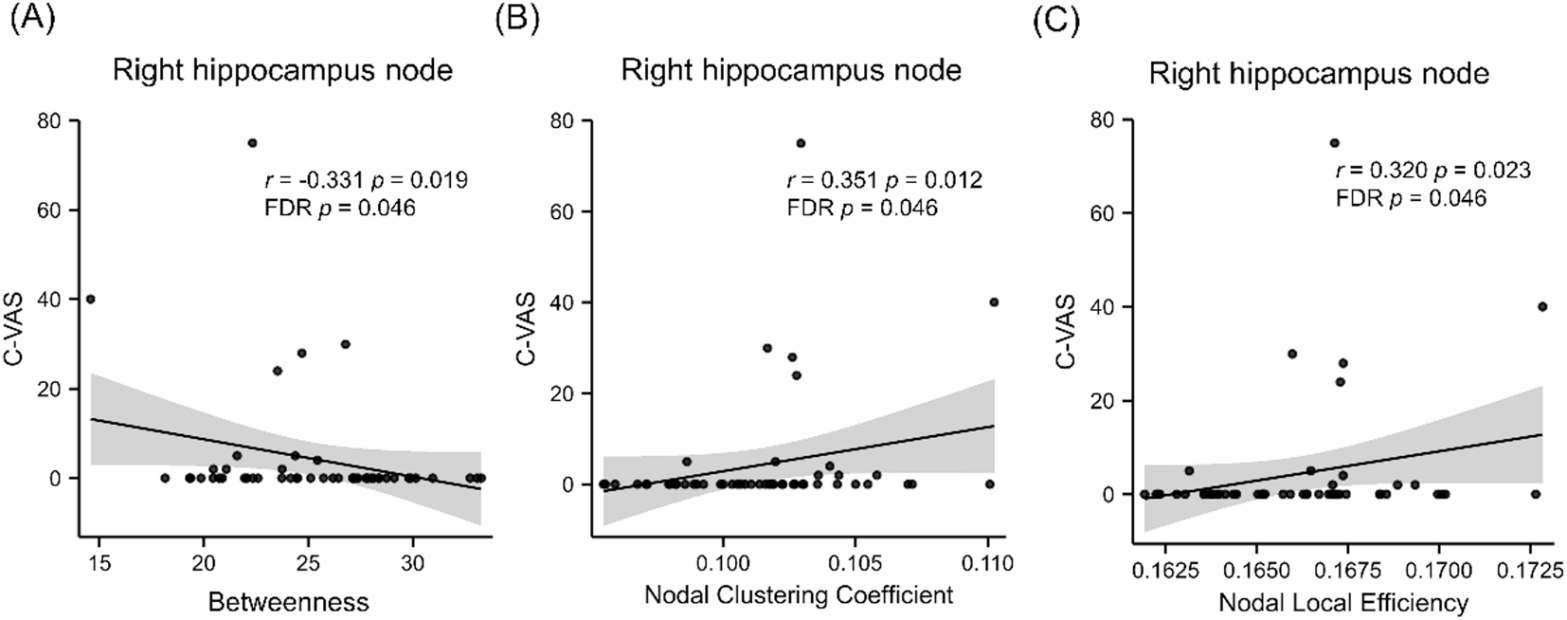
The relationships between nodal topological organization of right hippocampus and subjective craving in AD. (**A**) Scatter plot with a significantly negative correlation between nodal betweenness of right hippocampus and craving (C-VAS). (**B**) Scatter plot with a positive correlation between nodal clustering coefficient of right hippocampus and craving (C-VAS). (**C**) Scatter plot with a positive correlation between nodal local efficiency of right hippocampus and craving (C-VAS).

Spearman correlations were used to explore relationships between alcohol subjective craving and right hippocampus nodal topological properties. The results showed significantly negative correlations between lower right hippocampus nodal betweenness and self-reported craving, positive correlations between right hippocampus LE-nodal and positive correlations between CC-nodal and self-reported craving in the AD group (Fig. 5, Supplementary Table 6).

## Discussion

The traditional case-control study investigated potential neuro-substrates of AD with high genetic risk. At the WM microstructure level, cortical-striatal circuits exhibited disruptions in the FHP group relative to controls. In addition, at the nodal level, FHP showed nodal topological properties disturbances in right hippocampus, such as nodal betweenness, degree, efficiency and shortest path etc. Furthermore, the nodal topological properties of right hippocampus were significantly correlated with self-reported craving in AD.

Our research revealed that AD with FHP had a more severe level of dependence compared to FHN and HC. Our results are compatible with a previous study that FHP with more severe pathological features, which might indicate poor disease prognosis^14,48,49^. Observations have found impaired performance on problem-solving and other cognitive tasks in AUD, and these deficits tend to be more pronounced in patients with FHP^14,50,51^. Even substance-naïve offspring with a family history of AUD exhibit altered subcortical brain volumes structurally and abnormal executive-functioning and emotion-processing functionally compared with FHN peers^52^. Therefore, we conjecture that the observed WM changes in AD with FHP may reflect vulnerability and consequences of more severe use, which warrants further exploration. Variables as the presence of FHP may affect the effectiveness of interventions. Sub-analyses suggest that AD patients lacking a family history of alcohol problems may selectively benefit from nalmefene^8^. It has been repeatedly demonstrated that naltrexone helps reduce drinking in AD^53,54^. Further study suggested that patients with high levels of alcohol craving or a strong family history of AUD are more likely to benefit from naltrexone treatment^55^. The above findings indicate that FHP may be a marker of different clinical characteristics within AD patients. Therefore, neurobiological markers of AD with FHP may provide a theoretical basis for targeted treatment.

We found worse WM integrity of striatal circuits, specifically striatum-SMA and striatum-PCC, in FHP compared with HC. The results still have some implications even though they have not been corrected. The striatum plays a central role in the neuro-mechanism of addiction diseases^56,57^. The functional connectivity of cortical-striatal brain circuitry is heavily implicated in modulating alcohol-seeking and alcohol self-administration^58–60^. WM structures efficiently propagate neural signals between distinct cortical regions and improve brain functional connectivity^18^. The WM integrity of striatal circuits was related to clinical features and pathological behaviors in AD^28^. The diminished cortical-striatal structural connectivity, presumably reflecting ineffective prefrontal control and might lead to impulsive behaviors and incentive craving. Inhibitory control circuitry involves frontal regions, including the SMA and PCC^61^. The cortical-striatal circuitry may be the target circuit for prevention, especially for a subgroup of AD with FHP. Because WM integrity was influenced by both heritability risk and alcohol intoxication in FHP, the injuries were not apparent when alcohol use was controlled as a covariate in our study. It is possible that more severe abuse of alcohol after dependence in FHP further aggravates the damage to the WM integrity of reward systems.

We investigated the network topology of the hippocampal node was abnormally organized in AD, especially with FHP and found correlations with baseline craving. The hippocampus is important for the integration of emotion, reward and memory, and is particularly vulnerable to the neurotoxic effects of alcohol^62,63^. Alcohol exposure in humans results in impaired hippocampal-dependent learning and memory processes and may lead to pathological memory enhancement^64–66^. Moreover, it is not clear if the impaired hippocampal structure and function observed in AUD is a result of the excessive alcohol use or demonstrates a genetic predisposition to the development of heavy drinking. After controlling for covariates of alcohol use characteristics in our study, there was still damage to the topology organization in FHP compared to FHN, such as lower nodal betweenness, degree, efficiency and higher shortest path of right hippocampus. Brain structural networks can explain the structural basis of neural and physiological activities in the brain^67^and increased network connectivity is likely to play an important role in the system’s dynamics^68,69^. Lower nodal efficiency and an increased shortest path length in the right hippocampus suggest that the direct connections between the right hippocampus and more distal brain regions may be substantially compromised. In individuals with FHP, disrupted network topology might compromise hippocampal communication efficiency, induce aberrant activation, and generate dysfunctional connectivity^70^. These alterations collectively contribute to deficits in learning, pathological memory consolidation, and craving^71^. This aberrant organization may represent a neural substrate underlying the heightened vulnerability observed in FHP individuals for the development of AUD. In recent years, research on WM connectivity based on DTI has revealed that FHP have abnormalities in brain structural networks, such as overconnectivity or underconnectivity, and disrupted network topology properties^35^. We found that topological abnormalities are associated with enhanced subjective craving, suggesting that the disrupted hippocampal topology organization may be a neuroimaging marker of pathological reward enhancement. Physiotherapeutic approaches such as transcranial magnetic stimulation (TMS) may reduce craving and severity of alcohol use by modulating activity in cortico-subcortical circuits implicated in reduced frontal control and impaired downregulation^72–74^. The striatum and hippocampus regions and related circuits may be the therapeutic target for FHP subtype of AD.

Several limitations warrant consideration. First, the exclusive inclusion of male participants restricts generalizability, for gender-specific neurobiological responses to alcohol use merit further investigation. Second, the cross-sectional design limits our ability to infer causal relationships among abstinence duration, drinking behaviors, and DTI metrics. Third, residual confounding may persist due to unmeasured variables such as concomitant medications, comorbidities, and treatment engagement, despite our efforts to control for known confounders.

## Conclusion

In summary, abnormal WM nodal topological properties and disturbances of reward system were found in high genetic risk AD patients. The nodal topological properties of reward system specifically hippocampus was significantly correlated with self-reported craving in AD. Our findings could advance our understanding pathological mechanism of abnormal network topology organization of reward system. The neuroimaging neural substrates may indicate the therapeutic target for AD with high genetic risk.

## Supplementary Information

Below is the link to the electronic supplementary material.

Supplementary Material 1

## Data Availability

The datasets generated during and/or analysed during the current study are available from the corresponding author on reasonable request.
