# Supplementary material for: White matter neural substrates in alcohol dependence with genetic risk and their role in pathological reward process
Source: Sci Rep. 2025 Sep 26;15:33211. doi: 10.1038/s41598-025-18003-z (PMC12474950; doi:10.1038/s41598-025-18003-z)
Supplement: Supplementary file 1 — Supplementary Material 1 [file 41598_2025_18003_MOESM1_ESM.docx]

**White matter neural substrates in** **alcohol dependence with** **genetic risk and their role in pathological reward process**

Fei Wu^1†^, Guowei Wu^2†^, Ping Dong^1†^, Jiahui Deng^1^, Xuejiao Gao^1^, Peng Li^1^, Junliang Yuan^3†^, Hongqiang Sun ^1†^

^1^Peking University Sixth Hospital, Peking University Institute of Mental Health, NHC Key Laboratory of Mental Health (Peking University), National Clinical Research Center for Mental Disorders (Peking University Sixth Hospital), Peking University, Beijing, China, 100191.

^2^CAS Key Laboratory of Behavioral Science, Institute of Psychology, Chinese Academy of Sciences, Beijing, China.

^3^Department of Neurology, Peking University Sixth Hospital, Peking University Institute of Mental Health, NHC Key Laboratory of Mental Health (Peking University), National Clinical Research Center for Mental Disorders (Peking University Sixth Hospital), Peking University, Beijing, China

**Correspondence**

Junliang Yuan, Department of Neurology, Peking University Sixth Hospital, Peking University Institute of Mental Health, NHC Key Laboratory of Mental Health (Peking University), National Clinical Research Center for Mental Disorders (Peking University Sixth Hospital), Peking University, Beijing, 100191.

Email: junliangyuan@bjmu.edu.cn.

Hongqiang Sun, Peking University Sixth Hospital, Peking University Institute of Mental Health, NHC Key Laboratory of Mental Health (Peking University), National Clinical Research Center for Mental Disorders (Peking University Sixth Hospital), Peking University, Beijing, 100191.

Email: [sunhq@bjmu.edu.cn](mailto:sunhq@bjmu.edu.cn).

**Supplementary Table 1.** The difference of FA between FHP, FHN and HC in structural connections of striatal circuits.

|  | **FHP (n = 21)** | **FHN (n = 30)** | **HC (n = 25)** |  |  |  |
| --- | --- | --- | --- | --- | --- | --- |
|  | ***Mean (± SD)*** | ***Mean (± SD)*** | ***Mean (± SD)*** | ***F*** | ***P*** | ***FDR-BH P*** |
| ***FA*** |  |  |  |  |  |  |
| striatum-dlPFC L | 0.352(±0.161) | 0.357(±0.237) | 0.362(±0.218) | 1.096 | 0.340 | 0.476 |
| striatum-dlPFC R | 0.347 (±0.019) | 0.348(±0.018) | 0.353 (±0.021) | 0.810 | 0.449 | 0.571 |
| Striatum-mOFC L | 0.299(±0.022) | 0.309(±0.030) | 0.309 (±0.022) | 1.261 | 0.289 | 0.468 |
| Striatum-mOFC R | 0.302(±0.024) | 0.310(±0.031) | 0.309 (±0.022) | 0.671 | 0.514 | 0.599 |
| Striatum-SMA L | 0.401 (±0.022) | 0.409 (±0.025) | 0.420 (±0.024) | 4.212 | **0.019** | **0.088** |
|  | *diff = -0.008, p =0.671* | |  |  |  |  |
|  |  | *diff =-0.011, p =0.209* | |  |  |  |
|  | *diff = 0.020, p = 0.016* | | |  |  |  |
| Striatum-SMA R | 0.399 (±0.024) | 0.399 (±0.022) | 0.414 (±0.023) | 3.854 | **0.026** | **0.090** |
|  | *diff =-0.001, p =1.000* | |  |  |  |  |
|  |  | *diff =-0.016, p =0.039* | |  |  |  |
|  | *diff = -0.015, p = 0.047,* | | |  |  |  |
| Striatum-ACC L | 0.226 (±0.013) | 0.228 (±0.017) | 0.233 (±0.017) | 1.220 | 0.301 | 0.468 |
| Striatum-ACC R | 0.248 (±0.012) | 0.249 (±0.014) | 0.249 (±0.019) | 0.026 | 0.974 | 0.981 |
| Striatum-PCC L | 0.469 (±0.043) | 0.432 (±0.149) | 0.391 (±0.203) | 1.527 | 0.224 | 0.448 |
| Striatum-PCC R | 0.476 (±0.038) | 0.500 (±0.036) | 0.509 (±0.037) | 4.982 | **0.009** | **0.063** |
|  | *diff =0.024, p =0.073* | |  |  |  |  |
|  |  | *diff =-0.010, p =1.000* | |  |  |  |
|  | *diff = -0.034, p = 0.009* | | |  |  |  |
| Striatum-Amy L | 0.308 (±0.035) | 0.314 (±0.035) | 0.342 (±0.035) | 5.856 | **0.004** | **0.056** |
|  | *p = 1.000* | |  |  |  |  |
|  |  | *p =0.019* | |  |  |  |
|  | *p =0.008* | | |  |  |  |
| Striatum-Amy R | 0.351 (±0.028) | 0.357 (±0.027) | 0.358 (±0.024) | 2.216 | 0.116 | 0.270 |
| Striatum-Hippo L | 0.387 (±0.029) | 0.387 (±0.039) | 0.388 (±0.040) | 0.019 | 0.981 | 0.981 |
| Striatum-Hippo R | 0.377 (±0.017) | 0.385 (±0.023) | 0.392 (±0.026) | 2.678 | 0.075 | 0.210 |

The observed difference is calculated in turn as FHP-FHN, FHN-HC, FHP-HC and is assigned a p-value. Abbreviations: diff, observed difference; SD, standard deviation; FHP, family history positive; FHN, family history negative; HC, healthy controls; FA, fractional anisotropy; dlPFC, dorsolateral prefrontal cortex; mOFC, medial orbitofrontal cortex; SMA, striatum-supplementary motor area; ACC, anterior Cingulate Cortex; PCC, posterior cingulate cortex; Amy, amygdala; Hippo, hippocampus.

**Supplementary Table 2.** The difference of MD between FHP, FHN and HC in structural connections of striatal circuits.

|  | **FHP (n = 21)** | **FHN (n = 30)** | **HC (n = 25)** |  |  |  |
| --- | --- | --- | --- | --- | --- | --- |
|  | ***Mean (± SD)*** | ***Mean (± SD)*** | ***Mean (± SD)*** | ***F*** | ***P*** | ***FDR-BH P*** |
| ***MD*** |  |  |  |  |  |  |
| striatum-dlPFC L | 0.0084(±0.0004) | 0.0083(±0.0004) | 0.0082(±0.0003) | 2.100 | 0.130 | 0.202 |
| striatum-dlPFC R | 0.0085(±0.0005) | 0.0085(±0.0004) | 0.0082(±0.0003) | 4.096 | 0.021 | 0.073 |
|  | *p =1.000* | |  |  |  |  |
|  |  | *p =0.036* | |  |  |  |
|  | *p =0.061* | | |  |  |  |
| Striatum- mOFC L | 0.0087(±0.00046) | 0.0086(±0.00046) | 0.0085(±0.00040) | 1.794 | 0.174 | 0.243 |
| Striatum- mOFC R | 0.0086(±0.00038) | 0.0086(±0.00038) | 0.0084(±0.00031) | 0.689 | 0.505 | 0.642 |
| Striatum-SMA L | 0.0081(±0.0004) | 0.0081(±0.0005) | 0.0078(±0.0004) | 4.088 | **0.021** | **0.073** |
|  | *p =1.000* | |  |  |  |  |
|  |  | *p =0.086* | |  |  |  |
|  | *p = 0.029* | | |  |  |  |
| Striatum-SMA R | 0.0083(±0.0004) | 0.0083(±0.0004) | 0.0079(±0.0004)) | 6.957 | **0.002** | **0.014** |
|  | *p =1.000* | |  |  |  |  |
|  |  | *p =0.002* | |  |  |  |
|  | *p = 0.029* | | |  |  |  |
| Striatum-ACC L | 0.0011(±0.0001) | 0.0011(±0.0001) | 0.0009(±0.0001) | 15.224 | **<0.001** | **0.001** |
|  | *p =1.000* | |  |  |  |  |
|  |  | *p* <0.001 | |  |  |  |
|  | *p* <0.001 | | |  |  |  |
| Striatum-ACC R | 0.0012(±0.0001) | 0.0012(±0.0001) | 0.0012(±0.0001) | 0.026 | 0.974 | 0.974 |
| Striatum-PCC L | 0.0086(±0.0059) | 0.0077(±0.0026) | 0.0067(±0.0034) | 3.406 | **0.039** | **0.109** |
|  | *p =0.553* | |  |  |  |  |
|  |  | *p =0.463* | |  |  |  |
|  | *p = 0.033* | | |  |  |  |
| Striatum-PCC R | 0.0086(±0.0004) | 0.0086(±0.0012) | 0.0082(±0.0003) | 2.381 | 0.100 | 0.200 |
| Striatum-Amy L | 0.001(±0.000097) | 0.001(±0.000097) | 0.001(±0.000092) | 0.041 | 0.960 | 0.974 |
| Striatum-Amy R | 0.001(±0.00003) | 0.0009(±0.00006) | 0.001(±0.00005) | 0.343 | 0.710 | 0.828 |
| Striatum-Hippo L | 0.001(±0.0001) | 0.001(±0.0001) | 0.001(±0.0002) | 2.902 | 0.061 | 0.142 |
| Striatum-Hippo R | 0.0009(±0.00006) | 0.0009(±0.00005) | 0.0009(±0.00006) | 2.134 | 0.126 | 0.202 |

The observed difference is calculated in turn as FHP-FHN, FHN-HC, FHP-HC and is assigned a p-value. Abbreviations: SD, standard deviation; FHP, family history positive; FHN, family history negative; HC, healthy controls; MD, mean diffusivity; dlPFC, dorsolateral prefrontal cortex; mOFC, medial orbitofrontal cortex; SMA, striatum-supplementary motor area; ACC, anterior Cingulate Cortex; PCC, posterior cingulate cortex; Amy, amygdala; Hippo, hippocampus.

**Supplementary Table 3.** Left hippocampus nodal topological organizations among FHP, FHN and HC groups (covariate variables were controlled).

| **Node properties** | **FHP**  **(n = 21)** | **FHN**  **(n = 30)** | **HC**  **(n = 25)** | ***F*** | ***P*** |
| --- | --- | --- | --- | --- | --- |
| Between | 6.017±1.237 | 5.400±1.238 | 5.872±1.466 | 1.567 | 0.216 |
| Degree | 6.493±0.516 | 6.270±0.619 | 6.433±0.619 | 0.978 | 0.381 |
| CC-nodal | 0.157±0.005 | 0.157±0.006 | 0.155±0.007 | 0.713 | 0.494 |
| E-nodal | 0.144±0.003 | 0.142±0.003 | 0.142±0.005 | 1.100 | 0.338 |
| LE-nodal | 0.198±0.003 | 0.199±0.003 | 0.198±0.003 | 0.731 | 0.485 |
| Sp-nodal | 0.404±0.007 | 0.409±0.010 | 0.404±0.149 | 1.243 | 0.295 |

Abbreviations: FHP, family history positive; FHN, family history negative; HC, healthy controls; CC-nodal, Nodal clustering coefficient; E-nodal, Nodal efficiency; LE-nodal, Nodal local efficiency; Sp-nodal, Nodal Shortest path.

**Supplementary Table 4.** White matter tracts of striatal circuits in AD patients with FHP v. FHN (covariate variables were controlled).

|  | ***F*** | ***p*** | ***FDR p*** |  | ***F*** | ***p*** | ***FDR p*** |
| --- | --- | --- | --- | --- | --- | --- | --- |
| ***FA*** |  |  |  | ***MD*** |  |  |  |
| striatum-dlPFC L | 0.343 | 0.561 | 0.958 |  | 1.729 | 0.195 | 0.664 |
| striatum-dlPFC R | 0.058 | 0.801 | 0.958 |  | 0.261 | 0.612 | 0.779 |
| Striatum-mOFC L | 1.355 | 0.250 | 0.875 |  | 2.791 | 0.101 | 0.471 |
| Striatum-mOFC R | 0.562 | 0.457 | 0.958 |  | 0.521 | 0.474 | 0.664 |
| Striatum-SMA L | 1.854 | 0.180 | 0.840 |  | 1.136 | 0.292 | 0.664 |
| Striatum-SMA R | 0.129 | 0.721 | 0.958 |  | 0.072 | 0.790 | 0.922 |
| Striatum-ACC L | 0.006 | 0.937 | 0.958 |  | 1.024 | 0.317 | 0.664 |
| Striatum-ACC R | 0.014 | 0.907 | 0.958 |  | 0.014 | 0.907 | 0.977 |
| Striatum-PCC L | 0.003 | 0.958 | 0.958 |  | 0.543 | 0.465 | 0.664 |
| Striatum-PCC R | 5.190 | 0.027 | 0.378 |  | 0.593 | 0.445 | 0.664 |
| Striatum-Amy L | 0.264 | 0.610 | 0.958 |  | 0.000 | 1.000 | 1.000 |
| Striatum-Amy R | 0.632 | 0.434 | 0.958 |  | 0.956 | 0.333 | 0.664 |
| Striatum-Hippo L | 0.188 | 0.667 | 0.958 |  | 3.572 | 0.065 | 0.455 |
| Striatum-Hippo R | 2.298 | 0.136 | 0.840 |  | 5.343 | 0.025 | 0.350 |

Abbreviations: FA, fractional anisotropy; MD, mean diffusivity; FHP, family history positive; FHN, family history negative; dlPFC, dorsolateral prefrontal cortex; mOFC, medial orbitofrontal cortex; SMA, striatum-supplementary motor area; ACC, anterior Cingulate Cortex; PCC, posterior cingulate cortex; Amy, amygdala; Hippo, hippocampus.

**Supplementary Table 5.** White matter that exhibited altered nodal topological organization in AD with FHP v. AD with FHN (covariate variables were controlled).

| Nodal topological organization | Left hippocampus | | | | Right hippocampus | | |  |  |  |  | |
| --- | --- | --- | --- | --- | --- | --- | --- | --- | --- | --- | --- | --- |
|  |  |  |  |  |  |  |  | Left caudate | Left caudate | Left putamen | | Right putamen |
|  |  |  |  |  |  |  |  |  |  |  | |  |
|  | *F* | *p* value | FDR-BH *p* | *p* | | *p* value | FDR-BH *p* | *P* value | *p* value | *p* value | | *p* value |
| Betweenness | 2.114 | 0.153 | 0.480 | 0.084 | | **0.013** | **0.047** | 0.084 | 0.324 | 0.931 | | 0.822 |
| Degree | 0.653 | 0.423 | 0.635 | 0.547 | | **0.021** | **0.047** | 0.547 | 0.411 | 0.868 | | 0.455 |
| LE-nodal | 0.017 | 0.897 | 0.897 | 0.544 | | **0.039** | **0.047** | 0.544 | 0.236 | 0.490 | | 0.940 |
| CC-nodal | 1.418 | 0.240 | 0.480 | 0.240 | | 0.056 | 0.056 | 0.240 | 0.222 | 0.527 | | 0.951 |
| E-nodal | 0.017 | 0.897 | 0.897 | 0.072 | | **0.032** | **0.047** | 0.072 | 0.357 | 0.776 | | 0.485 |
| Sp-nodal | 1.917 | 0.173 | 0.480 | 0.073 | | **0.030** | **0.047** | 0.073 | 0.346 | 0.673 | | 0.502 |

Abbreviations: FHP, family history positive; FHN, family history negative; LE-nodal, Nodal local efficiency; CC-nodal, Nodal clustering coefficient; E-nodal, Nodal efficiency; Sp-nodal, Nodal Shortest path. Age, duration and severity (MAST) of AD were controlled as covariate variables in analysis

**Supplementary Table 6** Correlations analysis between nodal topological organization of right hippocampus and subjective craving

| Nodal topological organization | *r* | *p* | FDR *p* |
| --- | --- | --- | --- |
| Betweenness | -0.331 | **0.019** | **0.046** |
| Degree | -0.284 | 0.045 | 0.068 |
| LE-nodal | 0.320 | **0.023** | **0.046** |
| CC-nodal | 0.351 | **0.012** | **0.046** |
| E-nodal | -0.258 | 0.070 | 0.070 |
| Sp-nodal | 0.252 | 0.077 | 0.077 |

Abbreviations: LE-nodal, Nodal local efficiency; CC-nodal, Nodal clustering coefficient; E-nodal, Nodal efficiency; Sp-nodal, Nodal Shortest path.
